# Unveiling the anticancer effects of SGLT-2i: mechanisms and therapeutic potential

**DOI:** 10.3389/fphar.2024.1369352

**Published:** 2024-03-26

**Authors:** Min Sun, Jilei Sun, Wei Sun, Xiaonan Li, Zhe Wang, Liwei Sun, Yuehui Wang

**Affiliations:** ^1^ Department of Geriatrics, First Hospital, Jilin University, Changchun, China; ^2^ Changchun Traditional Chinese Medicine Hospital, Changchun, China; ^3^ First Affiliated Hospital of Jilin University, Changchun, China; ^4^ Research Center of Traditional Chinese Medicine, The Affiliated Hospital to Changchun University of Chinese Medicine, Changchun, China

**Keywords:** sodium-glucose cotransporter protein 2 inhibitors, anticancer, mechanisms, treatment, diabetes

## Abstract

Cancer and diabetes are significant diseases that pose a threat to human health. Their interconnection is complex, particularly when they coexist, often necessitating multiple therapeutic approaches to attain remission. Sodium-glucose cotransporter protein two inhibitors (SGLT-2i) emerged as a treatment for hyperglycemia, but subsequently exhibited noteworthy extra-glycemic properties, such as being registered for the treatment of heart failure and chronic kidney disease, especially with co-existing albuminuria, prompting its assessment as a potential treatment for various non-metabolic diseases. Considering its overall tolerability and established use in diabetes management, SGLT-2i may be a promising candidate for cancer therapy and as a supplementary component to conventional treatments. This narrative review aimed to examine the potential roles and mechanisms of SGLT-2i in the management of diverse types of cancer. Future investigations should focus on elucidating the antitumor efficacy of individual SGLT-2i in different cancer types and exploring the underlying mechanisms. Additionally, clinical trials to evaluate the safety and feasibility of incorporating SGLT-2i into the treatment regimen of specific cancer patients and determining appropriate dosage combinations with established antitumor agents would be of significant interest.

## 1 Introduction

Cancer, the primary contributor to global mortality, poses a significant impediment to the advancement of life expectancy. The prevalence of cancer is experiencing a notable surge, with an estimated 10 million fatalities predicted by 2020. The escalating burden of cancer incidence and mortality necessitates the implementation of comprehensive measures for cancer prevention and treatment to ensure effective global control (Sung et al., 2021). The development of novel pharmaceuticals for cancer control typically requires extensive characterization and clinical validation over an extended period. Alternatively, repurposing drugs with established anticancer properties may serve as a viable approach for advancing cancer treatment, particularly when these drugs with proven safety profiles are already routinely employed in clinical settings. Diabetes is emerging as a prevalent chronic, noncommunicable disease worldwide and is currently one of the most prevalent and rapidly growing global diseases. Projections indicate that the number of adults with diabetes will reach approximately 537 million by 2021, with a gradual increase that is expected to reach 783 million by 2045 (Sun et al., 2022). In 2021, diabetes was responsible for a significant mortality rate of 6.7 million individuals, thereby contributing to health expenditures of no less than $966 billion. Moreover, the condition imposes a substantial economic burden, primarily stemming from disparities in healthcare expenditures and limited access to treatment, particularly between developed and developing nations (Liyanage et al., 2015; da Rocha Fernandes et al., 2016; American Diabetes Association, 2018; Saeedi et al., 2019; Lin et al., 2020). Preventing and managing the progression of diabetes have emerged as prominent concerns in the global healthcare system. A multifaceted association exists between diabetes and certain types of cancer because tumors rely heavily on substantial quantities of glucose for glycolysis and energy generation to facilitate their metabolic processes, expansion, and propagation. Consequently, the suppression of glucose availability or hindrance of glycolysis effectively curtails cellular proliferation and tumor development (Yang et al., 2002; Chowdhury et al., 2016; Ling et al., 2020; Pearson-Stuttard et al., 2021).

**FIGURE 1:**
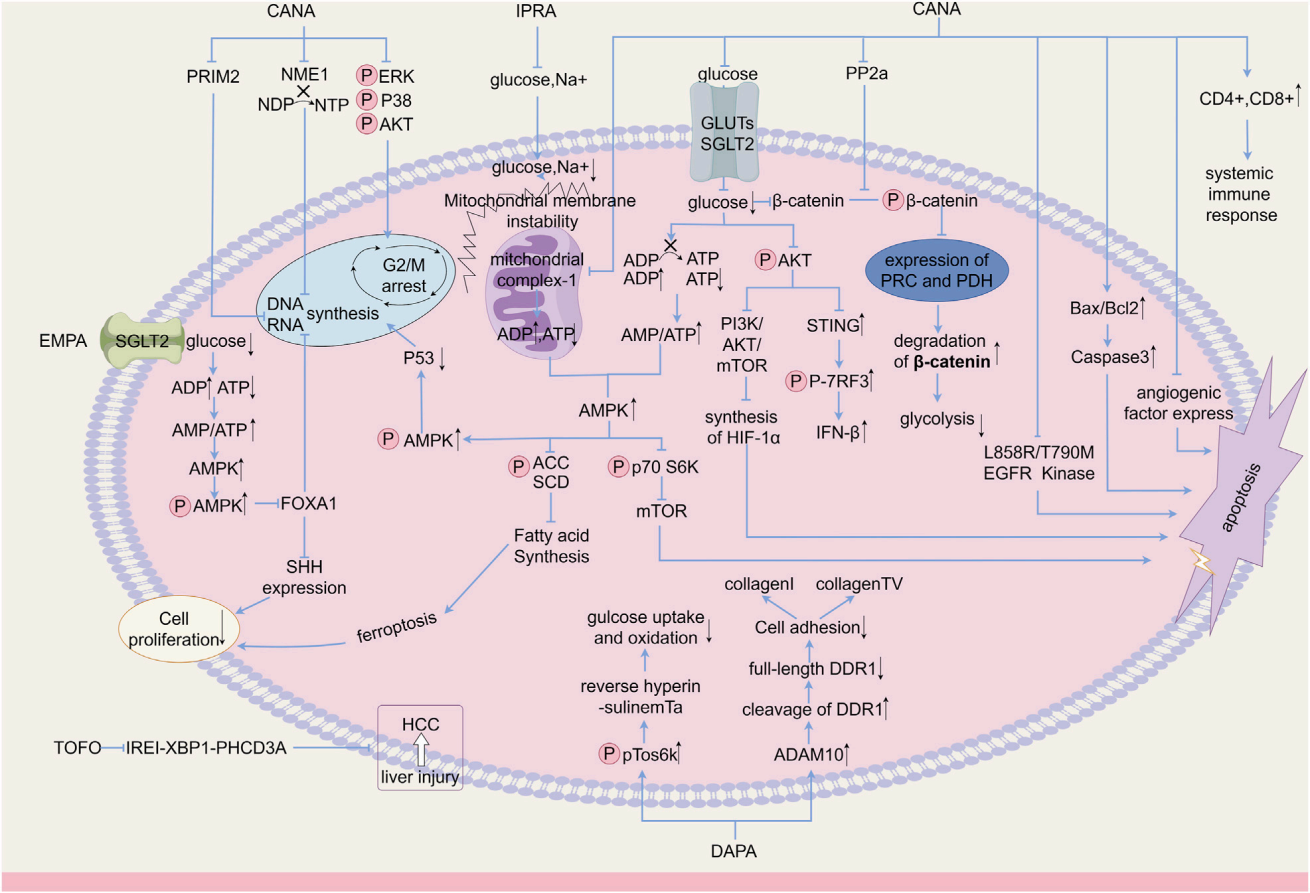
Anticancer mechanism of SGLT-2i.

SGLT-2i is a novel antidiabetic drug that effectively mitigates hyperglycemia by diminishing renal glucose reabsorption (Chao, 2014). In addition to their hypoglycemic effects, these inhibitors exhibit significant therapeutic potential for managing cardiovascular ailments in both diabetic and non-diabetic individuals. Moreover, their impeccable safety record in humans has prompted investigations into their viability as prospective treatments for various non-metabolic disorders (Rieg and Vallon, 2018). Recently, interest has increased in the antitumor effects of SGLT-2 inhibitors. Their use has been linked to lower risks of all-cause mortality, cancer-related mortality and new overall cancers( Dicembrini et al., 2019; Kato et al., 2019; García et al., 2021; Rokszin et al., 2021; Suissa et al., 2021; Ueda et al., 2022; Chinyama et al., 2023; Chung et al., 2023; Wang et al., 2024). Additionally, studies have shown the anticancer activity of SGLT-2i in various types of cancers including hepatocellular, pancreatic, prostate, colon, lung, and breast carcinomas (Saito et al., 2015; Scafoglio et al., 2015; Villani et al., 2016; Kuang et al., 2017; Obara et al., 2017; Tang et al., 2017; Kaji et al., 2018; Scafoglio et al., 2018; Shiba et al., 2018; Nasiri et al., 2019; Komatsu et al., 2020; Ding et al., 2023 ). Potential mechanisms include the reduction of glucose uptake by cancer cells, systemic glucose restriction, cell cycle arrest, DNA replication, regulation of multiple signaling pathways, and regulation of the expression of different genes and proteins. Given its general tolerability and routine use in diabetes management, SGLT-2i may be a good candidate for drug repurposing in cancer therapy, and could be used as an adjunct to conventional therapy. In this paper, we review the anticancer effects of SGLT-2i in different types of cancer reported in recent years and their possible mechanisms (Figure 1).

## 2 Expression of SGLTs

Overexpression of the glucose transporter GLUT1 in the SLC2A gene family is associated with increased cellular demand for glucose (Hung et al., 2019; Okada et al., 2020). Additionally, the expression of glucose transporters (GLUTs) and key enzymes such as hexokinase two and pyruvate kinase M2 are altered (Tohma et al., 2005; DeBerardinis et al., 2008; Hsu and Sabatini, 2008; Koppenol et al., 2011). In recent years, a second class of human glucose transporter proteins has been increasingly recognized within the SLC5 gene family, known as sodium-dependent glucose cotransporters (SGLTs) (Wright et al., 2011; Wright, 2013; Koepsell, 2017). SGLTs are widely acknowledged to play a crucial role in the absorption of glucose in the intestines and the reabsorption of glucose from the glomerular filtrate in the proximal tubules of the kidney (Wright et al., 2011; Sala-Rabanal et al., 2016; Ghezzi et al., 2018; Sala-Rabanal et al., 2018). Furthermore, apart from its presence in the small intestine and kidney, SGLT1 is expressed in the liver, lung, brain, and salivary glands, while SGLT2 is confined to the renal proximal tubule (Wright et al., 2011; Sabolic et al., 2012; Vrhovac et al., 2015; Ghezzi et al., 2017; Koepsell, 2017; Madunić et al., 2017). Recent investigations have revealed significant upregulation of SGLT2 in numerous tumors, including those affecting the liver (Kaji et al., 2018; Hung et al., 2019; Nakano et al., 2020; Ozutsumi et al., 2020; Li et al., 2021; Luo et al., 2021), pancreas (Scafoglio et al., 2015), prostate (Scafoglio et al., 2015; Villani et al., 2016), kidney (Kuang et al., 2017), lungs (Scafoglio et al., 2018), breast (Komatsu et al., 2020; Zhou et al., 2020), cervix (Xie et al., 2020), and colon (Saito et al., 2015; Okada et al., 2018; Okada et al., 2020), and that it plays a pivotal role in promoting cancer cell survival (Cangoz et al., 2013; Scafoglio et al., 2015; Kepe et al., 2018).

## 3 SGLT-2i species and their metabolic enzymes

Considering the observed protein expression and functional activity of SGLT2 in various tumor types, the glucose-lowering medication SGLT-2i has been investigated as a potential therapeutic agent for targeted treatment of specific cancers. Current research cannot determine the efficacy of certain SGLT-2i species in specific cancer types, but different types of SGLT-2i have different metabolic enzymes. For instance, dapagliflozin (DAPA) is metabolized and inactivated by uridine diphosphate-glucuronosyltransferase (UGT) family 1member A9 (UGT1A9), while empagliflozin (EMPA) is metabolized by UGT1A9 and other related isoforms of UGT family 2member B7, UGT family 1member A3, and UGT family 1member A8. Tofogliflozin, in contrast, undergoes metabolism by five different enzymes (CYP2C18, CYP3A4, CYP3A5, CYP4A11, and CYP4F3) before being inactivated and excreted from the body (Obermeier et al., 2010; Barron et al., 2016; Rieg and Vallon, 2018; Tokarz et al., 2018; Okada et al., 2020). Canagliflozin (CANA) is primarily metabolized to two pharmacologically inactive O-glucuronides (M7 and M5) by UGT1A9 and UGT2B4, respectively, whereas cytochrome P450 3A4 plays a minimal role in its metabolism (Devineni et al., 2015). Ipragliflozin is metabolized into multiple pharmacologically inactive metabolites predominantly via glucuronidation by UGT2B7, UGT2B4, UGT1A9, and UGT1A8 (Ferrannini et al., 2013)(Table1). Research has shown that DAPA has potential anticancer effects on colon cancer cells expressing SGLT2 but not UGT1A9 because cancer HCT116 cells express SGLT2 but not UGT1A9(Okada et al., 2018). Additional research is required to investigate the antitumor efficacy of different SGLT-2i types in different types of cancer. Specifically, clinical trials are required to evaluate the safety and feasibility of incorporating SGLT-2i into treatment protocols for patients with specific malignancies. Furthermore, these trials should aim to identify the optimal routes of administration for targeted delivery to specific tumor sites.

**TABLE 1:** Metabolic characteristics of different species of SGLT-2i.

Type of SGLT-2i	Duration	metabolic enzymes
DAPA	long-acting	UGT1A9
IPRA	long-acting	UGT1A9, UGT2B7, UGT2B4, UGT1A8
CANA	intermediate-acting	UGT1A9, UGT2B4
EMPA	intermediate-acting	UGT1A9, UGT2B7, UGT1A3, UGT1A8
TOFO	intermediate-acting	CYP2C18, CYP3A4, CYP3A5, CYP4A11, CYP4F3

## 4 SGLT-2i anticancer effects

### 4.1 Hepatocellular carcinoma

It is worth noting that the liver plays a crucial role in maintaining glucose homeostasis in the body, as hepatocytes not only consume glucose but also export it. An important characteristic that distinguishes tumors from normal tissues is the abnormal expression of glucose transporter proteins. Normal hepatocytes typically have high levels of bidirectional glucose transporters, notably GLUT2, which has a low affinity for glucose. Conversely, human liver tumors demonstrate elevated expression of GLUT1, GLUT2, and GLUT3 mRNAs (Su et al., 1990; Yamamoto et al., 1990). GLUT1 has a high affinity for glucose and facilitates asymmetric transport, leading to increased glucose uptake. Consequently, GLUT1 and GLUT3 upregulation substantially enhances glucose uptake in hepatocellular carcinoma (HCC) cells (Thorens, 1992; Grobholz et al., 1993; Xia et al., 2020). Furthermore, HCC cells also express SGLT-2. The anti-HCC effects of SGLT-2i have also been studied extensively (Table 2).

**TABLE 2:** Anti-hepatocellular carcinoma studies of SGLT-2i.

Studies	Research type	Cancer type	Type of SGLT-2i	Findings
Hung et al. (2019)	*in vivo* and *in vitro*	**Hepatocellular carcinoma**	CANA	**Event**: inhibited the growth of hepatocellular carcinoma
		Huh7, Hep3B cells tumor-bearing mice		**Mechanism**: impeded the activation of β-catenin signaling induced by glucose influx through the promotion of proteasomal degradation and inhibition of pp2a-mediated dephosphorylation of β-catenin
Kaji et al. (2018)	*in vivo* and *in vitro*	**Hepatocellular carcinoma**	CANA	**Event:** inhibited the proliferation of hepatocellular carcinoma cells, blocked the cell cycle to accelerate apoptosis, and attenuated the angiogenic activity of SGLT-2-expressing hepatocellular carcinoma **mechanism:** downregulated glycolytic metabolism through inhibiting extracellular signal-regulated kinase phosphorylation
		HepG2, Huh7 cells xenograft model mice		
Luo et al. (2021)	*in vivo* and *in vitro*	**Hepatocellular carcinoma**	CANA	**Event:** inhibited hypoxia-induced metastasis, angiogenesis, and metabolic reprogramming in hepatocellular carcinoma. **mechanism:** inhibited the expression of vascular endothelial growth factor, reduces epithelial-mesenchymal transition associated proteins and glycolysis-associated proteins, targets the AMPK/mTOR pathway
		HepG2, Hep3B, HCCLM3 cells		
		HepG2 xenograft mice		
Nakano et al. (2020)	*in vitro*	**Hepatocellular carcinoma**	CANA	**Event:** impeded the proliferation of HCC cells by modulating various metabolic pathways **mechanism:** altered mitochondrial oxidative phosphorylation and affect fatty acid metabolism
		Hep3B, Huh7 cells		
Jojima et al. (2019)	*in vivo* and *in vitro*	**Liver cancer** diabetes and NASH-HCC mouse	CANA	**Event:** inhibited liver tumorigenesis and was anti-fatty degenerative and anti-inflammatory **mechanism:** directly inhibited SGLT-2 and downregulated AFP mRNA and the expression of cyclin D and Cdk4 proteins
		HepG2 cells		
Abdel-Rafei et al. (2021)	*in vitro*	**Hepatocellular carcinoma**	CANA	**Event:** inhibited the clonal survival of HepG2 cells **mechanism:** downregulated glucose uptake, lactate release and regulation of endoplasmic reticulum stress-mediated autophagy
		HepG2 cells		
Kawaguchi et al. (2019)	*in vivo*	**Hepatocellular carcinoma**	CANA	**Event:** induced spontaneous regression of HCC in patients with cirrhosis combined with diabetes mellitus. **mechanism:** downregulated matrix metalloproteinase-8, angiopoietin-1, angiopoietin-2, prolactin and placental growth factor-aa
		Patients		
Li et al. (2021)	*in vivo* and *in vitro*	**Hepatocellular carcinoma**	DAPA	**Event:** mitigated hepatic steatosis **mechanism:** restored autophagy via the AMPK-mTOR pathway and promote the phosphorylation of ACC1 and upregulate the lipid β-oxidizing enzyme ACOX1
		HepG2 cells		

The WNT/β-catenin signaling pathway enhances aerobic glycolysis in cancer cells, resulting in increased pyruvate carboxylase and pyruvate dehydrogenase kinase one expression, ultimately leading to proteasome-mediated degradation of β-catenin (Reya et al., 2003; Zhan et al., 2017), which is a common genetic alteration in the development of HCC(Lee et al., 2012; Pate et al., 2014; Shibata and Aburatani, 2014). Hung et al. analyzed the clinical tumor samples from 216 patients diagnosed with HCC. Their findings revealed significant cytoplasmic and/or nuclear staining for β-catenin in liver tumors, while adjacent non-tumor sites did not exhibit such staining. In their investigation, using Huh7 and Hep3B cells as experimental models, CANA impeded the inward flow of glucose by inhibiting multiple GLUTs rather than SGLT2 alone. Previous studies also supported this conclusion, demonstrating that higher doses of CANA can affect not only SGLT2 but also other SGLTs and GLUTs, particularly GLUT1 (Nomura et al., 2010; Gurney et al., 2012). Additionally, CANA treatment effectively suppressed the survival and colony-forming capability of HCC in a dose-dependent manner (Hung et al., 2019). This inhibition was achieved by impeding the activation of β-catenin signaling induced by glucose influx, through the promotion of proteasomal degradation and inhibition of pp2a-mediated dephosphorylation of β-catenin. Consequently, HCC growth was inhibited, leading to an extended survival period for mice with tumors.

Protein kinase B (AKT), a crucial regulator of cancer cell survival, is involved in glycolysis regulation because its activation stimulates glucose uptake and aerobic glycolysis within cells (Elstrom et al., 2004). CANA showed dose-dependent antiproliferative effects on SGLT2-expressing Huh7 and HepG2 cells by downregulating glycolytic metabolism, including glucose uptake, lactate production, and intracellular ATP production (Kaji et al., 2018). The mechanism underlying this effect appears to involve the inhibition of extracellular signal-regulated kinase (ERK, p38, and AKT) phosphorylation and caspase3 cleavage, leading to G2/M cell cycle arrest and apoptosis. Additionally, oral administration of CANA significantly reduced the subcutaneous tumor load in xenograft hepatocellular carcinoma models derived from Huh7 and hepG2 cells. In this study, the oral administration of CANA resulted in a significant reduction in the subcutaneous tumor load of xenograft hepatocellular carcinoma tumors derived from Huh7 and HepG2 cells in BALB/c nude mice. CANA also reduced intra-tumoral angiogenesis. To further investigate these effects, *in vitro* experiments were conducted using human umbilical vein endothelial cells (HUVEC) co-cultured with Huh7 or HepG2 cells. CANA was found to inhibit the proliferation and tubule formation of HUVEC, indicating its ability to attenuate the pro-angiogenic activity of human HCC cells. In summary, CANA has the ability to block and inhibit angiogenic factors in hepatocellular carcinoma cells. Ozutsumi (Ozutsumi et al., 2020) discovered that the combined administration of CANA and teneligliptin, a dipeptidyl peptidase-4 inhibitor, effectively inhibited HCC cell growth and angiogenesis. Additionally, this treatment reduced oxidative stress and demonstrated a synergistic effect in the prevention of hepatocarcinogenesis. This finding was further supported by a clinical case study (Kawaguchi et al., 2019) in which a patient with cirrhosis and diabetes mellitus experienced spontaneous regression of HCC after taking CANA for 10 weeks. This regression was accompanied by a significant decrease in the expression of angiogenesis-related cytokines, including matrix metalloproteinase-8, vascular protein 1, vascular protein-2 prolactin, and placental growth factor-aa. Spontaneous tumor regression is a rare and poorly understood phenomenon that provides a new approach for tumor therapy.

Hypoxia is a commonly observed tumor microenvironment that induces various processes such as tumor metastasis, tumor angiogenesis, and glycolysis (Brown and Giaccia, 1998). The adaptive response to tumor hypoxia is primarily regulated by hypoxia-inducible factor 1 (HIF-1), a major promoter (Agani and Jiang, 2013). The biological activity of HIF-1 is influenced by the expression of HIF-1α, and the target genes regulated by HIF-1α have been identified to play a role in the systemic physiological responses of HCC to hypoxia, including glycolysis, metastasis, and angiogenesis (Guo et al., 2020). Luo et al. (Luo et al., 2021) demonstrated that CANA effectively inhibited hypoxia-induced metastasis, angiogenesis, and metabolic reprogramming in HCC. At the molecular level, the expression of vascular endothelial growth factor was inhibited, decreasing epithelial-mesenchymal transition-related and glycolysis-related proteins, and the synthesis of HIF-1α proteins was reduced without affecting their proteasomal degradation. Furthermore, evidence has demonstrated that CANA inhibits the AKT/mTOR pathway, which is crucial for HIF-1 transcription and translation.

Adenosine monophosphate-activated protein kinase (AMPK) and acetyl coenzyme A carboxylase (ACC) are sensors of intracellular ATP levels and modulators of β-oxidation. The role of AMPK in inducing G2/M blockade in HCC cells has been demonstrated through its modulation of the transcription factor p53 and the protein p21 (Lee W. et al., 2012; Sanli et al., 2014), as well as the inhibition of hepatic *de novo* lipogenesis and HCC proliferation via phosphorylation of ACC(Lally et al., 2019). ACAA1, an essential enzyme involved in the regulation of ketone body formation including fatty acid β-oxidation and 3-hydroxybutyric acid, plays a crucial role in promoting the development and progression of HCC(Liu et al., 2015; Yan et al., 2017). Nakano (Nakano et al., 2020) conducted a comprehensive investigation using multi-omics analysis of metabolomics and absolute quantitative proteomics to examine the effects of CANA on the proliferation and metabolic reprogramming of HCC cell lines. These findings suggest that CANA impedes HCC cell proliferation by modulating various metabolic pathways. Specifically, CANA upregulated nicotinamide adenine dinucleotide levels, thereby altering mitochondrial oxidative phosphorylation. Additionally, CANA downregulated ACAA1, affecting fatty acid metabolism and nucleoside diphosphate kinase 1 (NME1), and impacting purine and pyrimidine metabolism. Researchers have also observed the localization of SGLT-2 in the mitochondria of Hep3B and Huh7 cells, indicating that CANA may hinder the oxidative phosphorylation of AMPK. AMPK inhibition subsequently inhibits hepatic regeneration of adiposity and HCC proliferation through three distinct pathways: 1) phosphorylation of ACC, 2) downregulation of SCD, and 3) G2/M blockade in Hep3B cells. In a mouse model of HCC, the expression of nucleoside diphosphate kinases one and 2, which are responsible for the synthesis of nucleotide triphosphate from NDP, was upregulated (Hindupur et al., 2018; Puts et al., 2018). In Hep3B cells, CANA downregulates NME1 and upregulates NDP, while simultaneously downregulating the expression of DNA primase subunit 2 (PRIM2). This interference with DNA replication and mRNA transcription results in the inhibition of RNA and DNA synthesis. PRIM2 is a regulatory primase subunit that is involved in nucleotide formation, DNA replication, and transcription (Zerbe and Kuchta, 2002). Furthermore, a similar study demonstrated that DAPA effectively restored autophagy via the AMPK-mTOR pathway and mitigated hepatic steatosis in HepG2 cell models both *in vivo* and *in vitro*. Additionally, it promotes the phosphorylation of ACC1 and upregulates the lipid β-oxidizing enzyme 1 (ACOX1)(Li et al., 2021).

Abdel-Rafei et al. (2021) highlighted the efficacy of combining CANA and γ-IR in the treatment of HCC, as CANA enhances the antitumor potential of γ-irradiation (γ-IR) by inhibiting the clonal survival of HepG2 cells through downregulating glucose uptake, lactate release, and modulation of endoplasmic reticulum stress-mediated autophagy. Furthermore, CANA inhibits the signaling pathways involved in γ-IR-induced metabolic reprogramming and tumor progression, resulting in radioresistance and treatment failure (Abdel-Rafei et al., 2021). Specifically, CANA disrupts the communication between the PI3K/AKT/GSK-3β/mTOR and Wnt/β-catenin signaling pathways, enhances intracellular Ca^2+^-mediated apoptosis through the activation of caspase-12/caspase-3, downregulates the expression of p53 and Bcl-2, reduces endoplasmic reticulum stress-induced cytoprotective autophagy, and facilitates the interaction between autophagy and apoptosis in HepG2 cells.

Metabolic dysfunction associated steatohepatitis (MASH) is closely associated with type 2 diabetes and metabolic syndrome (Suzuki and Diehl, 2017; Tilg et al., 2017; Sheka et al., 2020; Rinella et al., 2023). The presence of a higher degree of steatosis, inflammation, and balloon degeneration in MASH is crucial in the development of cirrhosis and HCC and is strongly correlated with morbidity and mortality associated with liver disease (Haas et al., 2016; Taylor et al., 2020). Research has demonstrated that the combination of TOFO and pemafibrate, a selective PPARα modulator, holds therapeutic potential in halting the progression of MASH-associated HCC, enhancing HCC-related survival in STAM mice, reducing the incidence of liver tumors, and preventing liver injury through the inhibition of the IRE1-XBP1-PHLD3A pathway (Murakami et al., 2022). Two other *in vivo* studies demonstrated that the administration of TOFO improved the MASH-like liver phenotype in Western diet-fed melanocortin four receptor-deficient (Mc4r-KO) mice, which served as a human MASH mouse model. Additionally, TOFO treatment resulted in a reduction in the number of large tumors with a diameter of ≥2 mm and hindered the progression of MASH-associated liver tumors by ameliorating non-tumorigenic lesions (Shiba et al., 2018; Yoshioka et al., 2021). These findings are consistent with the conclusions drawn by Obara, who investigated the effects of TOFO on the development of MASH-associated liver tumorigenesis in C57BL/KsJ- + Lepr^db^/+Lepr^db^ obese and diabetic mice. The study revealed that TOFO significantly impedes the formation of pre-tumorigenic hepatic lesions, reduces hepatic steatosis, and alleviates hepatocellular balloon formation and inflammation (Obara et al., 2017). Jojima et al. demonstrated the inhibitory effects of CANA on liver tumorigenesis in mouse models of diabetes, MASH, and HCC. Continuous administration of CANA resulted in a significant reduction in the number of liver tumors compared with that in the control group. Additionally, the presence of glutamine synthetase-positive nodules was significantly reduced, and the mRNA expression of the HCC marker AFP was downregulated (Jojima et al., 2019). Flow cytometry analysis further confirmed that CANA decreased the percentage of HepG2 cells in the G2/M phase of the cell cycle, downregulated the expression of cyclin D and Cdk4, and increased the proportion of cells in the G0/1 phase. Additionally, CANA promoted apoptosis in HepG2 cells by activating caspase three and demonstrated anti-lipotropic and anti-inflammatory effects, thereby mitigating the advancement of MASH and preventing its progression to HCC. Similar findings have been made in clinical studies (Akuta et al., 2019; Takahashi et al., 2022 ). In essence, CANA induces cell cycle arrest and apoptosis in hepatocellular carcinoma, while also inhibiting tumor growth by directly inhibiting SGLT-2 in tumor cells. This indicates that the SGLT-2i attenuates the deterioration of MASH and prevents it from developing into HCC (Table 3).

**TABLE 3:** Precancer protection studies of SGLT-2i.

Studies	Research type	Cancer type	Type of SGLT-2i	Findings
Murakami et al. (2022)	*in vivo*	**Hepatocellular carcinoma**	TOFO	**Event:** enhanced HCC-related survival in STAM mice, reduced the incidence of liver tumors, and prevented liver injury **mechanism:** inhibited the IRE1- XBP1-PHLD3A pathway
		STAM mice		
Yoshioka et al. (2021)	*in vivo*	**Liver cancer**	TOFO	**Event:** improved the NASH-like liver phenotype and resulted in a reduction in the number of large tumors with a diameter of 2 mm or more
		Mc4r-KO mice and NASH mouse		
Obara et al. (2017)	*in vivo*	**Liver cancer**	TOFO	**Event:** impeded the formation of pre-tumorigenic hepatic lesions reduce hepatic steatosis, alleviated hepatocellular balloon formation and inflammation
		**C57BL/KsJ- + Lepr** ^***db***^ **/+Lepr** ^***db***^ **(*db/db*) mice**		
Shiba et al. (2018)	*in vivo*	**Liver cancer**	CANA	**Event:** reduced hepatic fibrosis, the number of liver tumors, and maximum tumor size **mechanism:** “healthy adipose expansion”
		Mc4r-KO mice		
Takahashi et al. (2022)	*in vivo*	**diabetes with** MASH patients	IPRA	**Event**: improved glycemic control, obesity, and hepatic outcomes, including liver fibrosis
Akuta et al. (2019)	*in vivo*	**patients with** MASH	CANA	**Event:** ameliorated liver steatosis, inflammation, hepatocyte ballooning, and fibrosis, as well as the abnormalities in liver function tests and glycemic control

### 4.2 Pancreatic and urinary tract cancer

Pancreatic cancer is one of the most lethal solid malignancies with poor prognosis and high mortality. Pancreatic cancer cells exhibit enhanced glycolysis and maintain rapid growth. CANA effectively inhibits the growth of pancreatic cancer in a dose-dependent manner, whether in cultured Capan-1 and PANC-1 cells or in PANC-1 derived tumor nude mice, and shows greater efficacy when used in combination with gemcitabine. Glucose metabolism is significantly inhibited in pancreatic cancer cells; specifically, glucose uptake and lactate production are reduced, and the mRNA levels of glycolysis-related genes (including glucose transporter-1 and lactate dehydrogenase A) are reduced. In addition, CANA induced early apoptosis of cancer cells and decreased the protein levels of PI3K, p-AKT, p-mTOR, and HIF-1α, indicating that CANA effectively inhibited the growth of pancreatic cancer by inhibiting glycolysis through the PI3K/AKT/mTOR signaling pathway (Xu et al., 2020).

ACC is a regulatory factor in fatty acid synthesis (Kahn et al., 2005; Lounis et al., 2017; Steinberg and Carling, 2019; Nakano et al., 2020). In a study evaluating the anticancer activity of SGLT-2 inhibitors (Villani et al., 2016), CANA inhibited the proliferation and clonal survival of prostate cancer cells (PC3, 22RV-1). A possible mechanism is that CANA strongly and dose-dependently inhibits mitochondrial complex I, causing cellular respiratory disorders, decreased ATP concentration, increased AMP/ATP ratio, and a rapid and significant increase in AMPK activity, leading to a significant increase in serine phosphorylation at position 79 of the ACC. However, blocking AMPK phosphorylation, inhibiting ACC, and overexpressing reduced nicotinamide adenine dinucleotide dehydrogenase subunit one complex I did not alter the inhibitory effect of CANA on cancer cell proliferation, indicating that CANA affects fatty acid synthesis by inhibiting respiration, supported by mitochondrial complex I, thereby limiting cancer cell proliferation. Furthermore, two clinical studies investigated the frequency of cancer in patients treated with SGLT2i, revealing a notable decrease in the likelihood of urothelial and hematologic malignancies alongside a lower incidence of cancer overall with SGLT2i therapy(Dicembrini et al., 2019; Rokszin et al., 2021 ).

In NSG xenograft mouse models, expression of the pancreatic and prostate cancer cell lines ASPC-1 and PC-3 was assessed (Scafoglio et al., 2015). The tumors were subsequently evaluated using microPET, *ex vivo* radioautography, and immunohistochemistry. These findings indicate that the specific novel radiotracer Me4FDG was distributed throughout the body, excluding the brain and bladder, and accumulated in crucial regions of the pancreas and prostate. Moreover, the oral administration of small doses of DAPA in mice resulted in a notable reduction of up to 40%–50% in prostate tumorigenesis. Additionally, oral administration of either high-dose CANA or DAPA significantly facilitated tumor necrosis by 70%–100% (Wright, 2020). CANA exhibited a notable reduction in tumor growth, suggesting that functional SGLT-2 is crucial for tumor survival. Furthermore, limited glucose diffusion within the tumor center may be attributed to its large size.

### 4.3 Breast cancer

Breast cancer is a highly significant cancer linked to type 2 diabetes and obesity (Lega and Lipscombe, 2020). A study conducted in Japan (Komatsu et al., 2020) investigated the expression of SGLT-2 in three distinct human breast cancer cell lines. SGLT-2 expression was found to be increased in highly estrogen-sensitive MCF-7 cells, whereas no such increase was observed in normal human mammary glands. Treatment with the SGLT-2 inhibitor IPRA resulted in a dose-dependent reduction in proliferation and DNA synthesis in MCF-7 cells. Notably, this effect was abolished when SGLT-2 was knocked down. The underlying mechanism may involve inhibition of glucose or sodium transport, leading to a decrease in intracellular sodium influx, hyperpolarization of the cell membrane, and instability of the mitochondrial membrane in MCF-7 cells. This results in an inhibitory effect on breast cancer cell proliferation.

A territory-wide study directly comparing the impact of SGLT2i and DPP4i on overall and predetermined cancer risk in a group of Asian patients demonstrated that DAPA is associated with a reduced risk of breast cancer (Chung et al., 2023). In agreement with a previous study (Komatsu et al., 2020), a comparative analysis of tumor and normal breast tissues acquired intraoperatively from 25 female patients with breast cancer revealed elevated mRNA and protein levels of SGLT-2 in breast cancer tissues compared with normal breast tissues. Furthermore, the administration of DAPA and CANA demonstrated dose-dependent inhibition of glucose transport in MCF-7 and ZR-75–1 human breast cancer cells (Zhou et al., 2020). This inhibition induced G1/G0 cell cycle arrest in MCF-7 cells, as well as suppressed proliferation and growth in human breast cancer cells and nude mouse MCF-7 cell xenografts. This mechanism potentially has a significant impact on glucose uptake reduction, oxidative phosphorylation inhibition in breast cancer cells, ATP production decrease, intracellular ATP concentration reduction, AMPK phosphorylation enhancement at the Thr172 locus, decrease in p70S6K phosphorylation, and mammalian rapamycin-targeted mTOR inhibition. This signaling pathway, in part, inhibits mTOR via AMPK activation, and is believed to be responsible for the inhibition of breast cancer cell proliferation, suppression of the G1 phase of the cell cycle, and induction of apoptosis (Zakikhani et al., 2012; Kennedy et al., 2020).

Glutamate dehydrogenase (GDH) activity has been linked to tumor cell adaptation to metabolic stress, and its overexpression has been recognized as an indicator of unfavorable cancer prognosis (Yang et al., 2014; Liu et al., 2015; Di Conza et al., 2019). Furthermore, it regulates redox homeostasis, promoting tumor growth (Jin et al., 2015). By utilizing CANA or DAPA in breast cancer cell lines characterized by high rates of aerobic glycolysis and glucose uptake (Papadopoli et al., 2021), the inhibition of cancer cell proliferation by SGLT-2i remained consistent regardless of the presence or absence of glucose. Therefore, the antiproliferative effect of the drug was not contingent on the level of glucose within the intracellular pathways. Further mechanistic studies demonstrated that CANA significantly reduced the levels of various intermediates of the citric acid cycle, including citrate, α-ketoglutarate, and succinate. Simultaneously, CANA increased the intracellular fluxes of glutamine, histidine, and lysine while notably inhibiting the activity of GDH. Consequently, this inhibition resulted in elevated intracellular glutamate levels and a decline in the intracellular concentrations of α-ketoglutarate, alanine, aspartic acid, and proline. These alterations ultimately led to a decrease in the tricarboxylic acid cycle activity and ATP production, thereby disrupting mitochondrial respiration and facilitating antiproliferative responses. Therefore, disruption of glutamine metabolism serves as a significant mechanism underlying the antiproliferative effects of CANA in breast cancer cells.

Hyperinsulinemia plays a pivotal role in the progression of obesity-associated tumors, and its reversal leads to the deceleration of tumor growth (Wang et al., 2018). In a mouse model of obesity-associated triple-negative breast cancer (E0771 tumor), glucose metabolism was found to be responsive to insulin, and the administration of excessive doses of DAPA and therapeutically relevant doses of CANA impeded cancer progression by attenuating glucose uptake and oxidation in E0771 tumors through the reversal of hyperinsulinemia (Nasiri et al., 2019).

### 4.4 Lung cancer

Lung cancer is a prominent contributor to cancer-related mortality worldwide, with non-small cell lung cancer (NSCLC) accounting for over 85% of all cases. The prognosis of NSCLC is notably unfavorable (Hirsch et al., 2017). Additionally, diabetes mellitus serves as an independent prognostic indicator of poor outcomes in individuals with lung cancer (Deng et al., 2019; Bi et al., 2020). Epidermal growth factor tyrosine kinase inhibitors (EGFR TKIs) exhibit remarkable clinical efficacy in advanced lung cancer. However, their effectiveness is significantly hindered by various mechanisms that facilitate drug resistance, primarily due to the presence of secondary EGFR T790M mutations that impede the binding of EGFR TKIs to receptor kinases, thereby nullifying their therapeutic impact. A study (Li et al., 2017) conducted on H1975 cells with EGFR L858R/T790M mutation revealed that CANA induces apoptosis in cancer cells through a mechanism independent of SGLT-2i or cellular glucose inflow. Additionally, CANA significantly reduced the anticancer activity of L858R/T790M EGFR kinase, thereby inhibiting the efficacy of EGFR TKIs in resistant lung cancer cells. Lung adenocarcinoma (LADC) and squamous cell carcinoma are the most prevalent histological subtypes of NSCLC. LADC primarily occurs in the terminal fine bronchioles and alveoli, which are not easily detectable using bronchoscopy. Furthermore, the identification of premalignant lesions associated with LADC is particularly challenging (Hirsch et al., 2017). Research findings indicate (Scafoglio et al., 2018) that SGLT-2 exhibits specific expression in premalignant and highly differentiated lung carcinomas, whereas precancerous and early lung adenocarcinomas predominantly rely on SGLT-2 for glucose transport into the tumor. In a mouse model, CANA effectively hindered tumor progression and retarded the development and growth of lung adenocarcinoma by restricting the supply of glucose to cancer cells. Administering CANA at an early stage of premalignant lung tumors can significantly diminish tumor burden and extend survival. Moreover, when combined with PET imaging, the ability to detect SGLT-dependent glucose transport *in vivo* enhances the response to SGLT-2i therapy.

An epidemiological analysis was conducted on a comprehensive dataset comprising a large, nationally representative sample of 24,915 patients newly diagnosed with NSCLC aged 66 years or older over a period of 2 years (Luo et al., 2023). This study aimed to investigate the association between SGLT-2i and cancer survival. The findings of this study revealed that the utilization of SGLT-2i was linked to enhanced overall survival rates among NSCLC patients with preexisting diabetes mellitus, irrespective of other confounding factors. An investigation (Yamamoto et al., 2021) was conducted on lung cancer tissues obtained from two elderly patients without diabetes who underwent segmental lung resection at Fukuoka University Hospital. This study revealed the presence of SGLT2 on the membranes of lung cancer cells. Subsequent treatment of the cancer cell lines with CANA resulted in a significant increase in the number of cells in the G0/G1 phase, and a concurrent dose-dependent decrease in the number of cells in the S phase. This effect did not induce apoptosis but rather led to cell cycle arrest, specifically from G1 to S entry. These findings suggested that CANA possesses anticancer properties on lung cancer cell lines by impeding cell cycle progression. Table 4.

**TABLE 4:** Anticancer studies of SGLT-2i.

Studies	Research type	Cancer type	Type of SGLT-2i	Findings
Xu et al. (2020)	*in vivo* and *in vitro*	**Pancreatic cancer**	CANA	**Event:** inhibited the growth of pancreatic cancer **mechanism:** inhibited glucose metabolism through the PI3K/AKT/mTOR signaling pathway
		Capan-1 and PANC-1 cells, PANC-1 derived tumor nude mice		
Villani et al. (2016)	*in vitro*	**Prostate cancer**	CANA	**Event:** inhibited the proliferation and clonal survival of the prostate
		PC3, 22RV-1 cells		**Mechanism**: affected fatty acid synthesis by inhibiting respiration supported by mitochondrial complex I
Rokszin et al. (2021)	*in vivo*	**Urinary tract cancer or hematological malignancy** patients	All SGLT-2i	**Event:** in patients on SGLT2i, the hazard of development of urinary tract cancer or hematological malignancy was half that of the hazard in patients taking DPP-4i
Dicembrini et al. (2019)	*in vivo*	**Bladder cancer** patients	All SGLT-2i	**Event:** DAPA treatment significantly reduces the incidence of bladder cancer
Wright (2020)	*in vivo*	**Pancreatic cancer and Prostate cancer**	CANA and DAPA	**Event:** reduced prostate tumorigenesis and facilitated tumor necrosis
		NSG xenograft mouse		
		ASPC-1 and PC-3 cells		
Komatsu et al. (2020)	*in vitro*	**Breast cancer**	IPRA	**Event:** attenuated breast cancer cell proliferation **mechanism:** induced cell membrane hyperpolarization and mitochondrial membrane instability
		MCF-7、MDA-MB-231 and KPL-1 cells		
Zhou et al. (2020)	*in vivo* and *in vitro*	**Breast cancer**	CANA and DAPA	**Event:** blocked human breast cancer cells proliferation and growth and induced cell apoptosis **mechanism:** induced AMPK-mediated cell cycle arrest and apoptosis
		MCF-7, SUM-131502, ZR-75–1 and T-47D cells		
		25 female patients xenograft breast cancer tumors in athymic nude mice		
Papadopoli et al. (2021)	*in vitro*	**Breast cancer**	CANA	**Event:** inhibited proliferation of breast cancer cells **mechanism:** reduced oxygen consumption and glutamine metabolism through the citric acid cycle
		SKBR3, BT-474, and MCF7 cell		
Chung et al. (2023)	*in vivo*	**Breast cancer** patients	DAPA	**Event:** DAPA is associated with a reduced risk of breast cancer
Nasiri et al. (2019)	*in vivo*	**Breast and Colon cancer**	DAPA	**Event:** impeded cancer progression **mechanism:** attenuated glucose uptake and oxidation through the reversal of hyperinsulinemia
		E0771 tumor mouse		
		MC38 homozygous mice		
Li et al. (2017)	*in vitro*	**Lung cancer**	CANA	**Event:** induced apoptosis in cancer cells **mechanism:** reduced the effectiveness of Anticancer activity of L858R/T790M EGFR kinase
		H1975 cells with the EGFR L858R/T790M mutation		
Scafoglio et al. (2018)	*in vivo*	**Lung cancer**	CANA	**Event:** hindered tumor progression and retards lung adenocarcinoma development and growth **mechanism**: restricted the supply of glucose to cancer cells
		Pre-cancerous and early LADC mice		
Luo et al. (2023)	*in vivo*	**Lung cancer**	CANA	**Event:** enhanced overall survival rates among NSCLC patients
		24,915 newly diagnosed NSCLC patients		
Yamamoto et al. (2021)	*in vivo* and *in vitro*	**Lung cancer** tissue from 2 non-diabetic lung cancer patients	CANA	**Event:** possessed anticancer properties against lung cancer cell lines **mechanism:** impeded cell cycle progression
		A549、H1975 and H520 cells		
Xie et al. (2020)	*in vivo* and *in vitro*	**Cervical cancer** a nude mouse model injected with HeLa cells	EMPA	**Event:** inhibited malignant proliferation and induced apoptosis **mechanism:** activated AMPK/FOXA1 pathway and inhibited SHH expression
Okada et al. (2020)	*in vitro*	**Colon cancer**	DAPA	**Event:** induced a loss of cell adhesion in cancer cells **mechanism:** inhibited cell adhesion to collagen types I and IV by enhancing ADAM10 activity, resulting in a loss of cell adhesion
		HCT116 cells		
Kuang et al. (2017)	*in vitro*	**Renal cancer**	DAPA	**Event:** inhibited cell growth **mechanism:** decreased glucose uptake, leading to downregulation of SGLT2, inhibited cell viability, arrested the G1 phase, promoted apoptosis
		RCC cell lines		
Wu et al. (2022)	*in vivo* and *in vitro*	**Osteosarcoma**	CANA	**Event:** inhibited osteosarcoma progression **mechanism:** activated the STING/IRF3/IFN-β pathway and induce immune cell infiltration
		K7M2 tumor-bearing mice osteosarcoma cells		
Okada et al. (2018)	*in vivo*	**Colon cancer** two colon cancer and type 2 diabetes mellitus patients	DAPA	**Event:** DAPA treatment may be linked to improved tumor markers in colon cancer patients

### 4.5 Osteosarcoma

Osteosarcoma, a rare bone malignancy with a poor prognosis, necessitates the development of novel therapeutic approaches. TRIM21, a ubiquitinated molecule, hinders cancer progression by facilitating the degradation of multiple molecules (Si et al., 2020; Zhou et al., 2021). Compared with osteoblast cell lines, osteosarcoma cells exhibit a notable decrease in TRIM21 protein expression. Immunoprecipitation demonstrated an interaction between SGLT2 and TRIM21, with TRIM21 suppressing SGLT2 degradation, whereas its overexpression led to the enhanced degradation of SGLT2. Consequently, upregulation of SGLT2 protein levels in osteosarcoma may be linked to diminished degradation of SGLT2 facilitated by TRIM21(Wu et al., 2022 ). STING mRNA and protein levels were upregulated in response to SGLT-2i in a dose-dependent manner. This effect is contingent on the expression of SGLT2, and the inhibition is nullified by the silencing of SGLT2. STING pathway agonist 2′3′-cGAMP effectively stimulates immune cells (Wu et al., 2013; Chandra et al., 2014). Furthermore, augmenting STING levels amplified the tumor immunotherapeutic effectiveness of cGAMP in mice (Lai et al., 2021). Conversely, AKT phosphorylation exhibited an inverse relationship with the STING pathway (Wu et al., 2019). CANA activated the STING/IRF3/IFN-β pathway in K7M2 tumor-bearing mice and osteosarcoma cells by impeding AKT phosphorylation levels (Wu et al., 2022), thereby synergistically inhibiting tumor growth when combined with 2′3′-cGAMP. Additionally, an increase in the population of circulating and splenic CD4^+^ and CD8^+^ lymphocytes was observed, suggesting the potential stimulation of a heightened systemic immune response, which induces immune cell infiltration to inhibit osteosarcoma progression.

### 4.6 Cervical cancer

Cervical cancer is a malignant tumor that affects women globally and ranks second in terms of occurrence (Wu et al., 2019). The development of this type of cancer involves a complex series of steps. The initiation of cervical cancer is attributed to the activation of specific pathways, including the well-known Wnt pathway, which facilitates the human papillomavirus (HPV)-induced transformation of germinal keratinized cells (Uren et al., 2005). The sonic hedgehog (Shh) pathway is widely expressed in various malignant tumors (Jeng et al., 2020) and plays a crucial role in promoting tumor cell proliferation, resistance to chemotherapy, and metastasis (Cochrane et al., 2015; Pak and Segal, 2016). Additionally, studies have demonstrated [9] that the combined effects of HPV oncoprotein and hedgehog signaling contribute to the acquisition of stem cell-like properties in cervical cancer cells. Xie et al. (Xie et al., 2020) investigated the antitumor effects of engeletin in a nude mouse model injected with HeLa cells. The results revealed that EMPA effectively suppressed the growth of mouse tumors in nude mice, inhibited the proliferation of cervical cancer cells, and induced apoptosis. Additionally, *in vitro* experiments using cervical cancer cell cultures demonstrated that EMPA hinders cancer cell migration and facilitates apoptosis. These effects were potentially mediated through activation of the AMPK/FOXA1 pathway, which impedes HeLa cell migration and promotes HeLa cell apoptosis. The relationship between Shh expression and overall survival time was determined using Kaplan–Meier survival analysis in patients with cervical cancer. These findings revealed that high Shh expression is associated with unfavorable overall survival. Additionally, EMPA activated AMPK phosphorylation and downregulated FOXA1 expression, leading to the inhibition of Shh expression. This suppressed malignant proliferation and induced apoptosis in cervical cancer cells.

### 4.7 Colon cancer

A study (Okada et al., 2020) investigated the effects of DAPA, EMPA, and TOFO in human colon cancer (HCT116), human hepatocellular carcinoma (HepG2), pancreatic carcinoma (PANC-1), and lung carcinoma (H1792) cells. The findings revealed that only DAPA induced loss of cell adhesion in cancer cells, with HCT116 cells exhibiting greater sensitivity to DAPA treatment than other cell types. Furthermore, the loss of cell adhesion was specific to DAPA and was not observed with EMPA or TOFO. The sensitivity of HCT116 cells to DAPA was influenced by SGLT-2 and UGT1A9 protein levels. Mechanistically, DAPA inhibited cell adhesion to collagen types I and IV by enhancing disintegrin and metalloproteinase structural domain protein 10 activity and promoting extracellular structural domain shedding of discoidin structural domain receptor family member 1 (DDR1), which results in a loss of cell adhesion and induces the dephosphorylation of Y792 tyrosine of DDR1, leading to a loss of substrate kinase activity downstream of DDR1.

In a clinical setting (Okada et al., 2018), levels of the colon cancer marker CEA were notably increased in two patients with both colon cancer and type 2 diabetes mellitus. Additionally, CEA levels in patients with colon cancer and type 2 diabetes mellitus, specifically those expressing SGLT2 but not UTG1A9, initially decreased after treatment with DAPA following radiotherapy, but subsequently increased upon discontinuation of DAPA. These findings suggest that DAPA treatment may be linked to improved tumor marker levels in patients with colon cancer. Therefore, for individuals with inconsistent levels of SGLT2 and UTG1A9, DAPA could potentially serve as an effective therapeutic agent against cancer.

Another study (Nasiri et al., 2019) demonstrated that AKT phosphorylation (pSer473) in tumors exhibited a temporary increase following a meal, with a slight delay compared with plasma insulin levels. Additionally, pThr389 p70 S6K levels were elevated in tumors during an oral glucose tolerance test conducted in MC38 homozygous mice with colon cancer. These findings suggest that the mechanism of action of the drug does not involve an increase in ketosis or a direct effect on tumor cell division. Instead, it indicates a significant alteration in tumor insulin signaling in response to normal physiological fluctuations in insulin concentration. Consequently, DAPA may impede the advancement of colon cancer associated with hyperinsulinemia by diminishing tumor glucose uptake and oxidation through the reversal of hyperinsulinemia, opening up an alternative mechanism for SGLT2-inhibited tumors.

### 4.8 Renal cancer

A study (Kuang et al., 2017) investigated the expression of SGLT2 in ACHN, A498, and CaKi-1 human renal cell carcinoma (RCC) cell lines, and Human Glandular KalliKrein-2 (HK-2) cells. The results revealed significantly higher mRNA expression level of SGLT2 in RCC cell lines than in HK-2 cells. Additionally, the SGLT2 inhibitor DAPA demonstrated a dose- and time-dependent inhibition of cell growth, with greater sensitivity observed in RCC cells than in HK-2 cells. This sensitivity may be attributed to the ability of DAPA to decrease glucose uptake in CaKi-1 cells, leading to the downregulation of SGLT2, inhibition of cell viability, arrest of the G1 phase, promotion of apoptosis, and reduction in malignant behavior. Furthermore, the administration of DAPA to nude mice resulted in a notable reduction in tumor size and a significant decrease in the expression of SGLT2, indicating a potential impact on tumor necrosis. Consequently, the functional manifestation of SGLT2 in renal cancer present novel prospects for its identification and management.

## 5 Conclusion

Despite substantial advancements in cancer therapy, cancer remains a prominent cause of mortality, ranking as the primary or secondary cause of death in 183 countries for individuals under the age of 70 (Sung et al., 2021). Consequently, there is a pressing need to explore more effective approaches. Hence, it is imperative to pursue more efficacious therapeutic or preventive strategies, particularly for specific cancer types characterized by rapid growth, metastasis, and treatment resistance. A burgeoning body of research investigates the utilization of selective SGLT-2i agents to impede cancer cell proliferation and induce apoptosis, a class of drugs whose intricate mechanisms of anticancer activity remain incompletely comprehended. However, the prospect of repurposing drugs currently used in the management of diabetes and heart failure for cancer therapy appears promising.

Given the increased susceptibility of patients with diabetes to cancer, it is imperative to consider modifying their glucose-lowering regimens to incorporate SGLT-2i, considering its potential anticancer properties. The selection of a specific SGLT-2i variant, the optimal treatment duration for assessing efficacy, and the appropriate antitumor drug combination necessitates immediate attention. Addressing these questions requires further investigation through preclinical and clinical studies to ascertain the prospective utilization and clinical advantages of these agents in oncology.

## 6 Perspectives

It is evident that numerous domains and potential targets exist for the anticancer properties of SGLT-2i. Despite unresolved inquiries, a substantial body of ongoing preclinical investigations and forthcoming clinical trials instill optimism about the prospective optimal utilization of this intriguing drug class in clinical oncology.
